# Recurrent Ischemic Strokes Due to Varicella-Zoster Virus (VZV) and Epstein-Barr Virus (EBV) Coinfection-Associated CNS Vasculitis in a Patient With AIDS: A Diagnostic and Therapeutic Challenge

**DOI:** 10.7759/cureus.90201

**Published:** 2025-08-16

**Authors:** Zhuo Luan, Aleksandr Drozdov, Jithendhar Kandimalla

**Affiliations:** 1 Neurology, Texas Tech University Health Sciences Center El Paso, El Paso, USA; 2 Radiology, Texas Tech University Health Sciences Center El Paso, El Paso, USA

**Keywords:** ebv encephalitis, hiv and aids, immunocompromised patient, recurrent ischemic stroke, vzv vasculopathy

## Abstract

We report an extremely rare case of CNS vasculitis caused by dual infection with varicella-zoster virus (VZV) and Epstein-Barr virus (EBV) in a man in his early 40s with newly diagnosed AIDS. He presented with seizures and encephalopathy, followed by recurrent multifocal strokes and MRI findings consistent with diffuse cerebral vasculitis. CSF analysis showed lymphocytic pleocytosis and tested positive for both VZV and EBV. Despite antiviral therapy and a brief course of corticosteroids, the patient experienced relapse and required retreatment. Clinical improvement was achieved with prolonged corticosteroid therapy combined with immune reconstitution via antiretroviral therapy. This case highlights the diagnostic challenges and potential for compounded vascular injury in dual herpesvirus CNS infection, emphasizing the importance of early recognition and the consideration of extended corticosteroid therapy in immunocompromised patients.

## Introduction

Cerebrovascular complications in patients with advanced HIV/AIDS are multifactorial, involving opportunistic infections, coagulopathies, cardioembolic events, and vasculitis. These complications may present subtly and can be easily overlooked. If not promptly recognized and treated, they can progress to severe outcomes, including stroke and potentially fatal consequences. Among opportunistic infections, herpesviruses, particularly varicella-zoster virus (VZV) and Epstein-Barr virus (EBV), can affect the CNS, causing a wide spectrum of neurological syndromes ranging from encephalitis and meningitis to vasculopathy and stroke [1]. While VZV is a well-established cause of CNS vasculitis and ischemic stroke, EBV-related CNS vasculitis remains rare but increasingly recognized [2]. Coinfection of the CNS with both viruses is exceedingly uncommon, especially in the setting of profound immunosuppression, and may contribute to a more severe inflammatory and neurovascular response [3].

VZV infection in immunocompromised individuals, particularly those with low CD4+ counts, often presents as a vasculopathy with multifocal ischemic infarcts. The virus is known to infect and replicate in cerebral arteries, triggering granulomatous arteritis and vessel wall necrosis [4]. EBV is primarily associated with lymphoproliferative disorders but has also been implicated in meningoencephalitis and vasculitis, particularly in patients with AIDS or transplant recipients [5]. The dual presence of VZV and EBV DNA in CSF raises concern for co-pathogenicity, in which synergistic viral and host immune mechanisms may accelerate endothelial damage and neuroinflammation [3].

This case describes a newly diagnosed AIDS patient with severe CD4+ depletion who presented with seizures and encephalopathy and was ultimately found to have VZV and EBV coinfection of the CNS. This led to recurrent ischemic strokes due to cerebral vasculitis and meningoencephalitis, with clinical improvement achieved after two courses of steroid therapy. We aim to highlight the diagnostic and therapeutic challenges posed by this rare coinfection and discuss the pathophysiology and clinical implications of herpesvirus-associated CNS vasculitis.

## Case presentation

A man in his early 40s with no prior medical history, who identified as homosexual, presented to another hospital on day 1 (first admission) with new-onset generalized tonic-clonic seizures and encephalopathy. According to the outside hospital records, a neurological examination revealed no focal deficits, but a brain MRI showed an 8 mm juxtacortical nodule in the left middle frontal gyrus with extensive vasogenic edema and leptomeningeal enhancement (images were not available, as they were performed at an outside hospital). EEG demonstrated mild diffuse slowing. Lumbar puncture (LP) revealed mild lymphocytic pleocytosis with mildly elevated protein and normal glucose (Table 1). CSF PCR was negative for HSV, and CSF cytology showed no evidence of malignancy. The patient was diagnosed with HIV-1 infection, with a viral load of 33,900 copies/mL (Table 2). Two stereotactic brain biopsies performed on days 7 and 14 revealed gliosis and focal inflammation, with no organisms or malignancy identified. No definitive etiology was determined. He was started on antiretroviral therapy (ART), levetiracetam, fluconazole, and broad-spectrum antibiotics for a presumed infectious brain process and was discharged home on day 22 with ART, fluconazole, and oral ciprofloxacin and metronidazole.

**Table 1:** CSF analysis Compared to the first admission, the CSF shows further increases in total nucleated cell count, protein level, and lymphocyte percentage. The meningitis panel was positive for VZV, and CSF PCR detected EBV. EBV, Epstein-Barr virus; VZV, varicella-zoster virus

CSF	First admission (outside facility)	Second admission	Reference
Total nucleated cells (/µL)	14	68	0-5
RBC (/µL)	2	3	0-5
Glucose (mg/dL)	49	38	40-70
Protein (mg/dL)	63	>300	12-60
Lymphocytes (%)	75	97	75
Meningitis panel	-	VZV positive	Negative
EBV PCR	-	Positive	Negative
CSF culture	Negative	Negative	Negative

**Table 2:** Blood lab work The patient tested positive for HIV-1. The HIV-1 viral load peaked during the second hospitalization but became undetectable at outpatient follow-up. The CD4 count was markedly low initially but improved at follow-up. Complement levels were low during the second admission.

Blood	First admission (outside facility)	Second admission	Follow-up at day 120	Reference
HIV ½ EIA	HIV-1 positive	HIV-1 positive	HIV-1 positive	Negative
HIV-1 viral load (copies/mL)	33,900	84,600	<20	<20
CD4 (cells/μL)	-	82	203	500-1,500
C3 (mg/dL)	-	80	-	88 and 201
C4 (mg/dL)	-	8	-	15-45

On day 48 (second admission), the patient was readmitted with five days of confusion. According to his family, he had appeared disoriented, with significantly reduced oral intake over the preceding five days and incomprehensible speech. On examination, he was encephalopathic without focal neurological deficits, including aphasia, motor weakness, or sensory loss. Empiric therapy was initiated with cefepime, vancomycin, fluconazole, metronidazole, and acyclovir due to concern for meningitis. Blood cultures were negative, and transthoracic echocardiography revealed no abnormalities.

**Figure 1:**
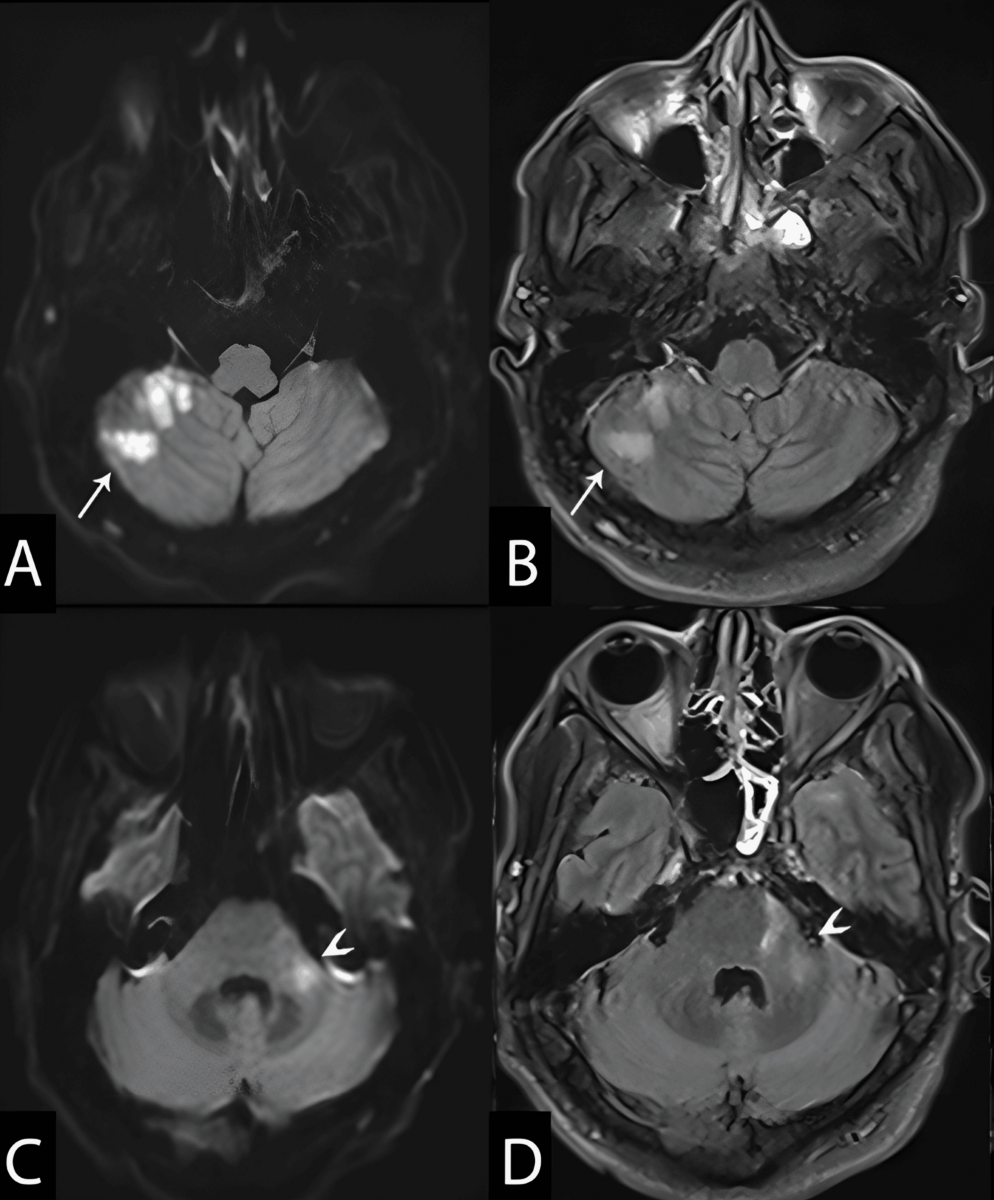
Brain MRI on day 48 All images are in the axial plane. Panels A and C show DWI b1000 images; panels B and D show T2 FLAIR images. The arrow indicates an acute ischemic infarction in the right cerebellar hemisphere, and the arrowhead marks an early subacute infarction in the left middle cerebellar peduncle. DWI, diffusion-weighted imaging; FLAIR, fluid-attenuated inversion recovery

**Figure 2:**
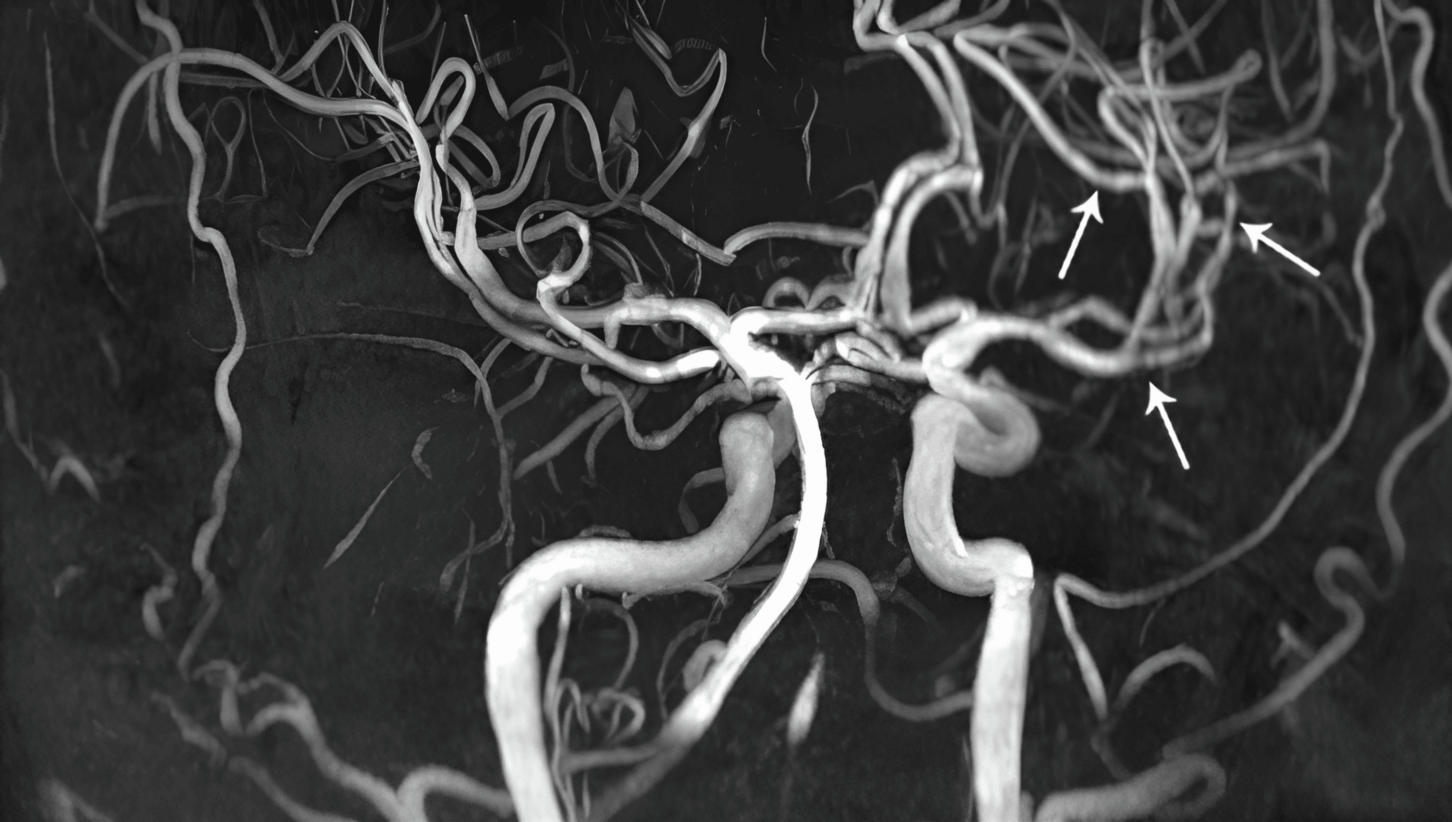
Brain MR angiography on day 48 MR angiography demonstrates multifocal luminal narrowing of the Circle of Willis arteries. Arrows indicate areas of narrowing in the M1-M3 segments of the right middle cerebral artery.

**Figure 3:**
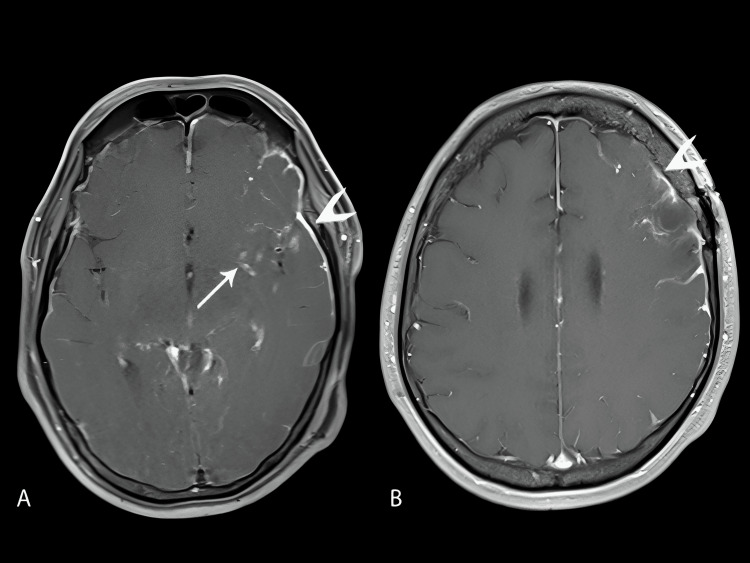
Additional MRI findings on day 48 Axial post-contrast T1-weighted images with fat saturation (A) and without fat saturation (B). The arrow shows abnormal enhancement of the perivascular spaces in the left basal ganglia (A), while the arrowhead highlights abnormal leptomeningeal enhancement along the left frontal lobe (A, B). A burr hole craniotomy and post-biopsy parenchymal defect are visible in the left middle frontal gyrus (B).

MRI revealed an acute right cerebellar infarct (Figure 1, arrows) and subacute infarcts in the left middle cerebellar peduncle (Figure 1, arrowheads). MR angiography demonstrated multiple areas of luminal narrowing (Figure 2), consistent with vasculitis. Additional MRI findings included leptomeningeal enhancement along the left frontal lobe and abnormal enhancement of the perivascular spaces in the left basal ganglia (Figure 3).

A repeat LP showed higher lymphocytic pleocytosis compared with the first admission, with markedly elevated protein and elevated lactate dehydrogenase (Table 1), consistent with worsening intracranial inflammation. Negative CSF cultures ruled out bacterial meningitis. A BioFire panel, a multiplex nested PCR assay with approximately 97% specificity, was performed to screen for multiple pathogens, including *Escherichia coli *K1, *Haemophilus influenzae*, *Listeria monocytogenes*, *Neisseria meningitidis*, *Streptococcus agalactiae*, *Streptococcus pneumoniae*, cytomegalovirus, *Cryptococcus neoformans/gattii*, enterovirus, HSV-1 and HSV-2, human parechovirus, and HHV-6. CSF was positive for VZV (via BioFire) and EBV (by PCR), with a concurrent HIV-1 viral load of 84,600 copies/mL and a low CD4 count (Table 2), consistent with VZV and EBV coinfection in the setting of AIDS. VZV IgM was negative, while EBV serologies indicated past infection (positive IgG against Epstein-Barr viral capsid antigen and nuclear antigen), suggesting reactivation due to immunosuppression. Complement levels were low (Table 2).

Because autoimmune vasculitis is typically associated with hypocomplementemia, further autoimmune workup was performed. ANA, anti-dsDNA, RF, ANCA (including p-ANCA and c-ANCA), and protease 3 antibody were all negative. Other infectious and autoimmune evaluations were unremarkable. The patient was diagnosed with VZV/EBV-associated meningoencephalitis and secondary cerebral vasculitis.

He was promptly treated with IV methylprednisolone (0.5 g BID) for three days, followed by an oral prednisone taper (10 mg daily for one week). IV acyclovir (10 mg/kg every eight hours) was continued for 14 days, followed by oral valacyclovir for one week. ART was maintained, and Bactrim prophylaxis was initiated. Following completion of IV methylprednisolone, the patient showed significant clinical improvement, becoming alert, awake, and able to follow commands. He was discharged home on day 54 with home IV infusion to complete the acyclovir course.

**Figure 4:**
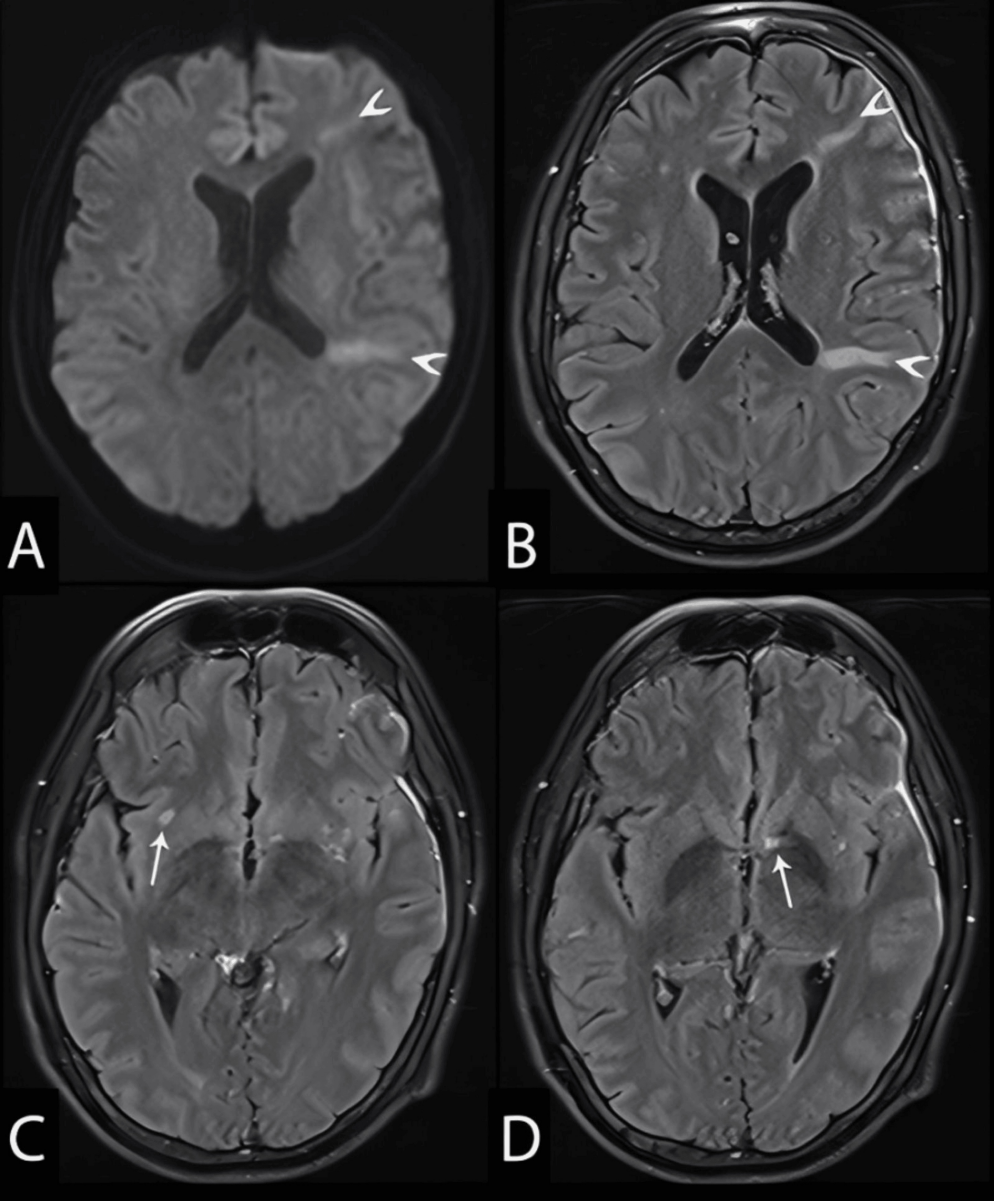
Brain MRI on day 65 Axial images. Panel A, DWI b = 1000; panels B-D, post-contrast T2 FLAIR images. New subacute ischemic infarctions are seen in the left frontal and left parietal corona radiata (arrowheads). Additionally, two focal ischemic infarcts have developed since the prior study: one in the right external capsule (arrow, panel C) and one in the left caudate head (arrow, panel D). Persistent leptomeningeal thickening along the left frontal lobe is similar to the previous study (B-D). DWI, diffusion-weighted imaging; FLAIR, fluid-attenuated inversion recovery

**Figure 5:**
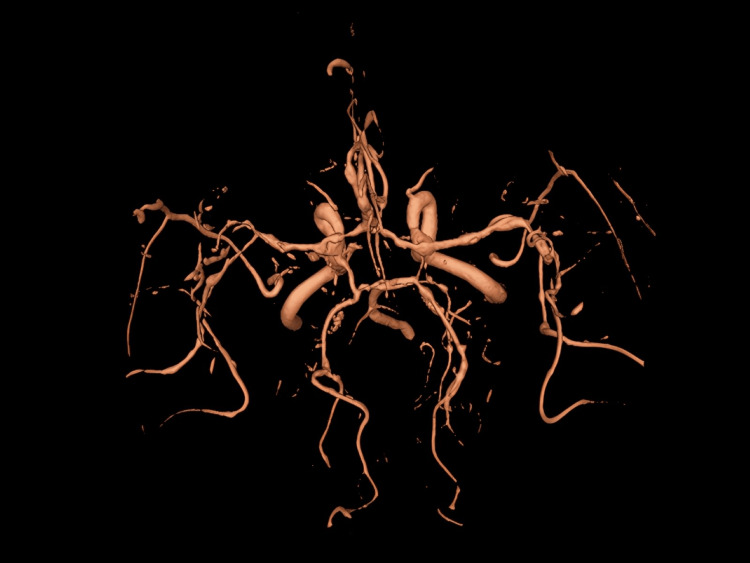
3D reconstruction from time-of-flight angiography on day 65 The reconstruction shows numerous areas of severe narrowing and post-stenotic dilatation throughout all segments of the anterior, middle, and posterior cerebral arteries. These findings are suggestive of vasculitis.

On day 65 (third admission), the patient returned with mild confusion. No seizures or focal neurological deficits were reported. MRI revealed the interval development of multiple ischemic infarcts in variable vascular territories (Figure 4). MRA demonstrated diffuse bilateral intracranial vasculitis, with multifocal moderate-to-severe luminal narrowing and post-stenotic dilation involving both the anterior and posterior circulations (Figure 5). EEG showed mild bifrontotemporal slowing.

He was retreated with IV methylprednisolone (0.5 g BID for three days) and continued on oral prednisone 10 mg daily, in addition to all his home medications. No additional acyclovir was administered, as he had already completed the antiviral treatment course. Given the worsening vasculitis, a repeat LP was recommended; however, the patient refused the procedure. Following completion of IV methylprednisolone, he improved significantly, becoming alert, awake, and able to follow commands, and was discharged home on day 73.

On day 80, the patient was seen in the neurology clinic. He was awake, alert, followed commands, answered questions appropriately, and had no focal neurological deficits. By day 120, his CD4 count had improved to 203/μL, and his HIV-1 RNA was undetectable (Table 2). Neurologically, he remained oriented and functionally independent. Plans were made to repeat imaging in the future.

## Discussion

Herpesviruses, particularly VZV and EBV, can reactivate under immunosuppressed conditions and invade the CNS, where they may cause a spectrum of neurological complications, including meningoencephalitis, vasculitis, and stroke [1]. In this case, both viruses were detected in the CSF of a patient with newly diagnosed AIDS who presented with seizures, encephalopathy, and recurrent multifocal strokes.

VZV is the most common viral cause of CNS vasculitis and ischemic stroke, particularly in immunocompromised individuals. The virus can infect the adventitia and media of cerebral arteries, leading to transmural inflammation, intimal proliferation, and granulomatous arteritis [4]. These vascular changes result in luminal narrowing, vessel wall irregularities, and thrombotic occlusion [6], as demonstrated in this patient’s MRI/MRA findings, which showed multifocal stenoses in both the anterior and posterior circulations. VZV vasculopathy often presents subacutely with cognitive changes, headache, and recurrent strokes. MRI typically reveals multifocal infarcts in both cortical and subcortical regions, while MRA or vessel wall imaging may demonstrate vessel wall thickening, enhancement, and narrowing, all of which were observed in this patient. CSF findings usually include lymphocytic pleocytosis, elevated protein, and normal or mildly decreased glucose. Diagnosis is confirmed by detecting VZV DNA in the CSF via PCR, as in this case. Prompt antiviral treatment with IV acyclovir and adjunctive corticosteroids is standard, although the optimal duration of therapy remains unclear [7,8].

EBV CNS disease in AIDS patients is most commonly associated with primary CNS lymphoma, but it may also present as meningoencephalitis or vasculitis, particularly in individuals with advanced immunosuppression [1]. EBV can infect endothelial cells and B-lymphocytes, contributing to CNS inflammation and vascular injury [9]. Although EBV-related vasculitis is rare, it has been reported in transplant recipients and other immunocompromised hosts, often presenting with encephalopathy and multifocal infarcts [2,5]. In this case, EBV DNA was detected by qualitative PCR in the CSF, raising suspicion for concurrent EBV-mediated CNS inflammation. Serologies indicated past exposure; however, reactivation can occur in immunocompromised patients. EBV reactivation may amplify the local immune response, increase vascular endothelial injury, and potentiate the effects of other viruses. Although EBV is also associated with lymphoma, prior brain biopsy and CSF cytology were negative, making CNS lymphoma unlikely in this patient.

Coinfection of the CNS with both VZV and EBV is exceedingly rare, even in immunocompromised individuals. It could result in a compounded inflammatory process involving the meninges and/or cerebral vasculature [3], thereby increasing the risk of stroke and encephalopathy, as seen in this patient. The patient’s progressive clinical course despite adequate antiviral treatment and a single course of corticosteroids may reflect additive or synergistic effects of both viruses on vascular inflammation. Complement levels were low in this patient, suggesting that coinfection could lead to intensified complement activation, enhanced immune complex-mediated damage, and more severe or recurrent CNS vasculitis.

Moreover, the worsening of vasculitis on imaging following completion of acyclovir and a single course of corticosteroids suggests that antiviral therapy combined with a short steroid course may be insufficient in severe immunosuppression with dual viral infection. In this patient, corticosteroids were reinitiated to suppress persistent inflammation, resulting in excellent clinical recovery. This suggests that extended corticosteroid therapy, along with ART-induced immune reconstitution (as evidenced by a rise in CD4 count >200 cells/μL and undetectable HIV viral load at follow-up), may be critical for clinical stabilization. However, the patient’s initial improvement followed by relapse underscores the potential need for extended corticosteroid therapy and consideration of maintenance immunomodulatory treatment. Lee et al. reported successful treatment of EBV-associated CNS vasculitis with rituximab [10]; however, the use of rituximab in HIV-associated CNS vasculitis remains unexplored.

Diagnosing viral vasculitis in immunocompromised patients can be challenging, as imaging findings often overlap with other etiologies such as opportunistic infections, malignancy, and autoimmune processes. In this case, negative fungal and mycobacterial cultures, normal CSF cytology, and stereotactic brain biopsy ruled out malignancy and abscess, while imaging and CSF findings supported an inflammatory process. Vessel wall imaging played a critical role in detecting cerebral vasculitis, particularly in smaller distal branches that may appear normal on conventional MRA.

Treatment strategies for VZV-associated vasculitis typically include high-dose IV acyclovir and corticosteroids to control inflammation [7]. There are no established antiviral therapies for EBV; management is largely supportive and focuses on immune reconstitution with ART in HIV-positive individuals. Currently, there are no definitive guidelines or robust clinical trials supporting the use of aspirin in VZV-induced vasculitic stroke, largely due to the rarity of the condition. Aspirin may be considered in patients with concomitant traditional vascular risk factors; however, this was not the case in our patient, and aspirin was not initiated. In this case, the patient’s CD4 count increased to over 200/μL with clinical improvement, suggesting that ART combined with extended corticosteroid therapy may be sufficient for disease control.

This case illustrates the importance of early ART initiation with extended corticosteroid therapy, vigilant neurological monitoring, and maintaining a high index of suspicion for multi-pathogen involvement in AIDS-related CNS disease.

## Conclusions

Recurrent strokes in patients with AIDS should prompt consideration of viral vasculitis, especially when imaging shows vessel wall enhancement and multifocal infarcts. Although rare, dual reactivation of VZV and EBV can occur in severely immunocompromised individuals and may lead to more aggressive CNS inflammation. Early recognition through comprehensive viral testing, along with timely initiation of antiviral therapy and extended corticosteroid treatment, is critical for clinical stabilization and improved outcomes.
